# New Trends in Aryl Hydrocarbon Receptor Biology

**DOI:** 10.3389/fcell.2016.00045

**Published:** 2016-05-11

**Authors:** Sonia Mulero-Navarro, Pedro M. Fernandez-Salguero

**Affiliations:** Departamento de Bioquímica y Biología Molecular y Genética, Facultad de Ciencias, Universidad de ExtremaduraBadajoz, Spain

**Keywords:** Aryl hydrocarbon receptor, differentiation, endogenous ligand, epigenetics, pluripotency, therapeutic target, transposons

## Abstract

Traditionally considered as a critical intermediate in the toxic and carcinogenic response to dioxin (2,3,7,8-tetrachlorodibenzo-*p*-dioxin, TCDD), the Aryl hydrocarbon/Dioxin receptor (AhR) has proven to be also an important regulator of cell physiology and organ homeostasis. AhR has become an interesting and actual area of research mainly boosted by a significant number of recent studies analyzing its contribution to the proper functioning of the immune, hepatic, cardiovascular, vascular and reproductive systems. At the cellular level, AhR establishes functional interactions with signaling pathways governing cell proliferation and cell cycle, cell morphology, cell adhesion and cell migration. Two exciting new aspects in AhR biology deal with its implication in the control of cell differentiation and its more than likely involvement in cell pluripotency and stemness. In fact, it is possible that AhR could help modulate the balance between differentiation and pluripotency in normal and transformed tumor cells. At the molecular level, AhR regulates an increasingly large array of physiologically relevant genes either by traditional transcription-dependent mechanisms or by unforeseen processes involving genomic insulators, chromatin dynamics and the transcription of mobile genetic elements. AhR is also closely related to epigenetics, not only from the point of view of target gene expression but also with respect to its own regulation by promoter methylation. It is reasonable to consider that deregulation of these many functions could have a causative role, or at least contribute to, human disease. Consequently, several laboratories have proposed that AhR could be a valuable tool as diagnostic marker and/or therapeutic target in human pathologies. An additional point of interest is the possibility of regulating AhR activity by endogenous non-toxic low weight molecules agonist or antagonist molecules that could be present or included in the diet. In this review, we will address these molecular and functional features of AhR biology within physiological and pathological contexts.

## Introduction

Since the early 90's, the Aryl hydrocarbon/Dioxin receptor (AhR) has been defined as an environmental-sensor PAS (Period [Per]-Aryl hydrocarbon receptor nuclear translocator [Arnt]-Single minded [Sim]) protein structurally included within the class I of basic helix-loop-helix transcriptional regulators (Burbach et al., 1992; Ema et al., 1992; Swanson and Bradfield, 1993; Hankinson, 1995) with major roles in xenobiotic-induced toxicity and carcinogenicity (Boffetta et al., 2011; Pohjanvirta, 2012). A distinctive feature of AhR is that, up to date, is among the only member of PAS proteins known to be activated by ligands (Bersten et al., 2013). Among the ample diversity of environmental xenobiotics, polycyclic aromatic hydrocarbons have been extensively studied as AhR ligands, with a particularly intense investigation on the human carcinogen 2,3,7,8-tetrachlorodibenzo-p-dioxin (Dioxin, TCDD; Kerkvliet et al., 2002). The molecular pathway leading to AhR activation by exogenous ligands (e.g., TCDD) has been extensively studied. Unliganded AhR resides in the cytosolic compartment of the cell bound to a molecular chaperone complex that at least contains two molecules of Hsp90, XAP2 and p23. Upon ligand binding, the receptor translocates to the nucleus and heterodimerizes with the class II bHLH protein ARNT/HIF1β (Aryl hydrocarbon receptor nuclear translocator/Hypoxia-inducible factor 1β) (Swanson and Bradfield, 1993; Swanson et al., 1995; Crews, 1998; Whitlock, 1999; Nebert and Dalton, 2006). Many reports have described at the molecular level the mechanism of AhR-dependent transcription (Pohjanvirta, 2012). Despite some controversy about the existence of an endogenous ligand(-s) that binds AhR in the absence of xenobiotics, once activated, AhR translocates to the cell nucleus to interact with ARNT in order to form a functional AhR-ARNT heterodimer (Reyes et al., 1992). This heterodimer binds to a set of co-activators and/or co-repressors and the resulting complex will recognize a consensus XRE binding site (xenobiotic responsive element; 5′-GCGTG-3′) located in the upstream r(Mimura et al., 1999)egulatory region of target genes (e.g., cytochromes P450 such as CYP1A1) (Hankinson, 1995, 2005; Whitlock, 1999). Following transcriptional regulation, the AhR-ARNT heterodimer is disassembled from DNA and AhR is transported to the cytosol for proteosomal degradation (Davarinos and Pollenz, 1999; Ma and Baldwin, 2000; Santiago-Josefat et al., 2001). Moreover, the AhR repressor (*AhRR)* has been identified as a target gene of AhR, providing a novel mechanism of feedback inhibition of AhR function in that a transcription factor directly induces the expression of its repressor through binding to its cognate regulatory sequence located in the promoter of the target gene. This regulatory circuit involves the activation of AhR by xenobiotics in order to induce *AhRR* expression, which, in turn, will compete with AhR for ARNT heterodimerization. As AhRR-ARNT heterodimers are transcriptionally inactive, AhR-dependent gene expression results abolished by reduced AhR-ARNT recruitment to the XRE sites located in the promoter of responsive genes (Mimura et al., 1999).

Based on its functions as a mediator of the cellular response to xenobiotics, initial human epidemiological studies alerted to health authorities about the pathological effects likely attributable to AhR-mediated activity including alterations in the immune system, lipid metabolism, epithelial integrity, porphyria, liver damage, thymic involution and cancer (Fingerhut et al., 1991; Flesch-Janys et al., 1995). However, the generation of transgenic mouse models led to a remarkable number of findings supporting that AhR plays important physiological and homeostatic roles in major organs and tissues. Among others, AhR-null mice (*AhR-/-*) showed developmental defects in the hepatic, hematopoietic, cardiovascular and immune systems (Fernandez-Salguero et al., 1995, 1997; Schmidt et al., 1996; Mimura et al., 1997; Lahvis et al., 2000, 2005). Recent evidences suggest the existence of endogenous ligands for AhR (Nguyen and Bradfield, 2008) triggering differential responses that could lead to cell-type specific AhR-dependent effects. Additionally, AhR levels are regulated by the proteasome (Davarinos and Pollenz, 1999; Ma and Baldwin, 2000), and proteasome inhibition, in absence of exogenous ligands, can activate AhR transcription (Santiago-Josefat et al., 2001; Santiago-Josefat and Fernandez-Salguero, 2003). The physiological relevance of AhR is further supported by its evolutionary conservation across metazoan phyla (Qin and Powell-Coffman, 2004; Qin et al., 2006; Williams et al., 2014). It is remarkable that invertebrate isoforms of AhR such as *D. melanogaster* Spineless (Ss) does not have detoxifying functions but it is instead required for eye, leg and wing development (Céspedes et al., 2010). In *C. elegans*, AHR-1 appears essential for neuronal differentiation and migration (Qin and Powell-Coffman, 2004; Qin et al., 2006). The current view in the field is that xenobiotic-dependent AhR functions in fact represent an adaptive mechanism overlapping its physiologically conserved roles.

In this review, we will discuss novel functions and regulatory mechanisms of AhR that have improved our knowledge on this receptor in the physiological and pathological contexts. The first stages in AhR research involved great efforts to discover and to characterize its many roles in mediating the toxicity of dioxins and related compounds, with some initial studies identifying homeostatic and developmental processes requiring its activity. Recent investigations are discovering new and exciting insights into AhR biology dealing with cell differentiation and pluripotency, chromatin dynamics, activation of mobile genetic elements, proliferation, epidermal barrier function and immune regulation (Safe et al., 2013). An additional aspect that is been recently considered is the potential beneficial effect of including natural AhR ligands in the diet, with the aim to maintain the endogenous AhR expression and activation that supports normal cell functioning. Current and future work will determine whether AhR intervention represents a useful tool to treat certain human diseases.

## New mechanisms of AhR regulation

**Figure 1:**
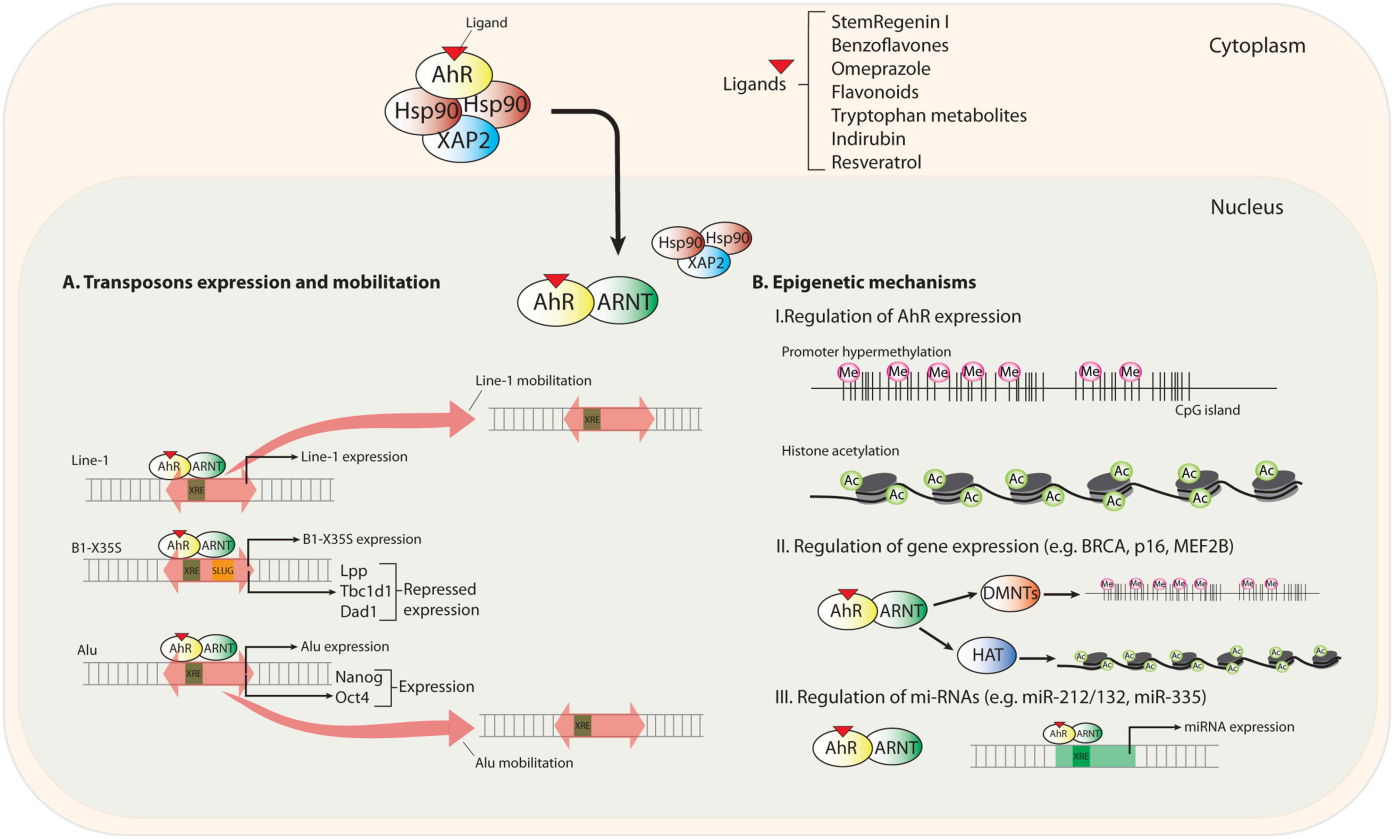
**Scheme summarizing new mechanisms of AhR regulation**. Upon novel (non-toxic) ligand binding, AhR translocates to the nucleus and heterodimerizes with its partner protein ARNT. **(A)** Transposable elements (TEs) located in upstream promoter and enhancer sequences contain transcription factor binding sites (TFBSs) for gene regulation. AhR recognizes consensus XRE binding elements (xenobiotic responsive element; 5′-GCGTG-3′) in *LINE-1, B1-X35S*, and *Al*u TEs. AhR-mediated regulation triggers the expression and mobilization of these TEs. **(B)** Epigenetics mechanisms regulating AhR expression and the status of its target genes and microRNAs.

In the first part of this section, we will discuss recently identified mechanisms through which AhR modulates gene expression and that differ from classical transcriptional regulation. We will focus on the activation of mobile genetic elements as unexpected intermediates in the AhR-dependent control of gene expression. In a second part, we will analyze the impact of epigenetics in regulating AhR expression and in how AhR could influence the epigenetic status of target genes (Figure 1).

### AhR in the control of gene expression through mobile genetic elements

Transposable elements are no longer considered useless DNA without a function in the genome. Accumulating evidence supports an important role for these mobile molecules in development and disease (Beck et al., 2011; Hancks and Kazazian, 2012; Solyom and Kazazian, 2012). Among them, retrotransposons of the murine *SINE* (short interspersed nuclear elements) and human *Alu* subfamilies account for close to 13% of their respective genomes and, more importantly, they are highly abundant in intronic and upstream promoter regions of target genes (Lander et al., 2001; Versteeg et al., 2003; Kriegs et al., 2007; de Koning et al., 2011). An intriguing feature of transposable elements is their ability to carry binding sites for well-known transcription factors, including OCT4 (POU5F1), CTCF, SOX2, NANOG, p53 and ESR1 (Wang et al., 2007; Bourque et al., 2008; Kunarso et al., 2010). Studies in mouse and human embryonic stem cells estimate that transposable elements provide nearly 25% of the binding sites for OCT4 and NANOG transcription factors, suggesting that transposons have an active influence in determining gene expression patterns (Wissing et al., 2012; Friedli et al., 2014; Elbarbary et al., 2016). An intense effort is currently underway to identify mobile genetic elements whose activation could regulate cell functions under normal and pathological conditions (Bennett et al., 2008; Fort et al., 2014; Hung et al., 2015), as observed for the retrotransposon-mediated oncogenic activation in human hepatocellular carcinoma (Shukla et al., 2013).

Notably, the dioxin receptor is functionally connected to the regulation of transposable elements. Early work has revealed that AhR activation by the xenobiotic compound benzo-*a*-pyrene (BaP) induced the expression of *LINE-1* (long interspersed nuclear element-1) retrotransposons in human cell lines from cervical carcinoma (HeLa) and microvascular endothelium (HMEC), and in mouse cells from smooth muscle (mVSMC) and embryonic kidney (mK4). Interestingly, when the prototypical AhR ligand TCDD (2,3,7,8-tetrachlorodibenzo-*p*-dioxin) was used, *LINE-1* induction was only observed in HeLa cells, suggesting that *LINE-1* retrotransposons may be modulated by cell type-specific mechanisms of AhR activation (Teneng et al., 2007). A latter study analyzing the *LINE-1*^*RP*^ human retrotransposon revealed that its effects on cell proliferation and differentiation could be recapitulated by the activation of endogenous *LINE-1* elements by the AhR ligand BaP (Ramos et al., 2011). Human exposure to low concentrations of environmental carcinogens seems to contribute to tumorigenesis (Lauber et al., 2004). In this regard, carcinogens present in broiled meet seem to regulate *LINE-1* retrotransposition by an AhR-dependent process. Nanomolar concentrations of food-borne 2-amino-1-methyl-6-phenylimidazo[4,5-b]pyridine (PhIP) and 2-amino-3,8-dimethyl-imidazo [4,5-f]quinoxaline (MeIQx) were shown to induce *LINE-1* mobilization by a mechanism that requires AhR, MAPK (mitogen-activated protein kinase) and C/EBPβ (CCAAT enhancer binding protein-β), suggesting a link between carcinogens, LINE-1 elements and AhR (Okudaira et al., 2013). Surprisingly, however, the tryptophan photoproduct and non-carcinogenic endogenous AhR ligand FICZ (6 formylindolo[*3,2-b*]carbazole) induced *LINE-1* retrotransposition in human hepatocellular carcinoma HuH-7 cells by a mechanism requiring ARNT and MAPK but not AhR (Okudaira et al., 2010). One possible explanation for these results is that bHLH/PAS proteins other than AhR modulate the epigenetic status of active *LINE-1* elements, so that genome reorganization by FICZ-induced ARNT-mediated *LINE-1* transposition gives the cell an advantage for survival (Okudaira et al., 2010). Although it appears very likely that AhR modulates *LINE-1* activation, future studies are needed to clarify the molecular mechanisms and the signaling pathways involved.

AhR can regulate gene expression through non-autonomous *B1-SINE* and *Alu* retroelements. We initially identified a novel *B1-SINE* retrotransposon (named *B1-X35S*), widely represented in the mouse genome, and that was particularly enriched in upstream promoter regions (Roman et al., 2008). *B1-X35S* was a conserved sequence harboring binding sites for AhR (XRE) and for the E-box binding proteins Snail and Slug/Snai2. Importantly, the AhR/ARNT heterodimer and Slug/Snai2 were co-recruited under basal and ligand-induced conditions to *B1-X35S* elements located in the promoter of developmental and differentiation-related genes *Lpp* (cell-adhesion related lipoma preferred partner), *Tbc1d1* (Obesity risk gene), and *Dad1* (Defender against cell death 1) (Roman et al., 2008). From a functional point of view, ligand mediated AhR activation induced the repression of these target genes in mouse hepatocellular carcinoma Hepa-1 cells whereas mutation of AhR and Slug binding sites restored basal gene expression (Román et al., 2011a). Later studies investigating the molecular mechanisms involved in the repressive activity of *B1-X35S* revealed that this element has a potent autonomous insulator activity *in vitro* in Hepa-1 cells and *in vivo* in zebrafish (Román et al., 2011b). A central observation from this study was that AhR-dependent repression of *Lpp, Tbc1d1* and *Dad1* (also *Cabin 1* and *Rtl1*) required the transcription of *B1-X35S* by a switch mechanism involving RNA polymerase III and RNA polymerase II. That is, the repression of target genes does not take place by a direct effect of AhR on the transcription of their mRNAs (by RNA polymerase II) but rather by a mechanism triggered by the non-coding RNA transcripts produced from the *B1-X35S* retrotransposon (Román et al., 2011a). The location of *B1-X35S* in between the upstream enhancer and the promoter of target genes (e.g., *Lpp, Tbc1d1, Dad1, Cabin 1*, and *Rtl1*), that is responsible for its insulator activity, was suggestive of the existence of a local change in chromatin structure flanking the retrotransposon (Román et al., 2011b). *B1-X35S* established a chromatin barrier upstream and downstream of its gene-specific position that resulted in an AhR/Slug-dependent enrichment in me3H3K9 and me3H3K27 repressive marks. We proposed from this study that AhR-dependent transcription of *B1-X35S* triggers a repressive mechanism by acting as an insulator and heterochromatin barrier. Nevertheless, we could not determine at that time the precise mechanism through which transposon-derived non-coding RNA transcripts could repress gene expression.

Experiments performed in human embryonic carcinoma NTERA-2 cells helped answer such question. Differentiation of NTERA-2 cells with retinoic acid (RA) produced a marked increase in AHR protein levels that was followed by its nuclear translocation and transcriptional activation (Morales-Hernández et al., 2016). The search for AHR targets led to the identification of pluripotency inducing genes *NANOG* and *OCT4* as those having an *Alu* element in their promoters that contain active XRE and E-box sites for AHR and Slug/Snai2 binding. Notably, repression of *NANOG* and *OCT4* mRNAs required AHR binding to the *Alu* element and its transcription by RNA polymerase III, thus producing *Alu*-derived non-coding RNA transcripts that will have a prominent role in the repressive process. RNA sequencing (RNAseq) allowed the identification of *NANOG-Alu* and *OCT4-Alu* derived transcripts. The analysis of the microRNA machinery of NTERA-2 cells using RISC (RNA-induced silencing complex) inhibitors, dominant-negative forms of Microprocessor components DROSHA and DGCR8 and AGO-2 driven RNA immunoprecipitation (RIP), led us to propose a model in which *NANOG-Alu* derived transcripts were processed through the miRNA pathway to generate small non-coding RNAs complementary to the 3′UTR region (unstranslated region) of *NANOG* and *OCT4*. Such complementarity would eventually reduce the mRNA levels of both genes in *cis* and *trans*, respectively. Consistently, NTERA-2 cells engineered to stably express a small-hairpin RNA for AHR (sh-AHR) had a basal undifferentiated status, did not respond to RA-induced differentiation and had a marked reduction in non-coding RNA transcripts from *NANOG* and *OCT4 Alus* (Morales-Hernández et al., 2016). This study also revealed that AHR induces differentiation in human carcinoma cells and that its downmodulation promotes undifferentiation. This functional interaction of AHR with mobile genetic elements in the control of gene expression represents a novel mechanism that could contribute to establish gene expression programs required for cellular reprogramming and for the maintenance of an undifferentiated status.

### Epigenetic mechanisms

Epigenetic modifications refer to dynamic changes introduced in genes by specialized enzymes, which do not alter their DNA nucleotide sequence itself, but instead modify how they are transcribed. DNA methylation, post-translational modification of histones and chromatin remodeling are the main epigenetic modifications so far described, and alteration of these mechanisms is known to be associated with different human pathologies. Initial cancer driving events involve genome-wide epigenetic silencing of transcription factors that disrupt gene networks, specifically, those targeted by EZH2/polycomb, p53, c-Myc, and AhR (Locke et al., 2015). For instance, in MCF7 human breast cancer cells, AhR activation was linked to silencing of BRCA1 by recruitment of DNA methyltransferases to its promoter region (Papoutsis et al., 2015). In human keratinocytes, AhR was reported to induce silencing and hypermethylation of the CDKN2A (p16) tumor suppressor gene and inhibition of p53 signaling (Frauenstein et al., 2013). However, within this epigenetic context, more efforts are necessary to unravel the precise connection between epigenetic modifications and transcriptional regulation of AhR, either through the receptor itself or through its target genes.

Regarding the regulation of *AhR* expression, we described for the first time that *AhR* is an epigenetically regulated gene (Mulero-Navarro et al., 2006). The human *AHR* promoter has CpG islands susceptible to be methylated and that could result in gene silencing. In particular, acute lymphoblastic leukemia (ALL) REH and chronic myeloid leukemia K562 cell lines had high levels of *AhR* promoter methylation and did not express detectable levels of receptor under basal conditions (Mulero-Navarro et al., 2006). Furthermore, *AhR* was methylated in 33% of primary human ALL tumors, suggesting that *AhR* silencing in this tumor type could hamper the likely tumor suppressor activity of this receptor. Interestingly, the frequency of *AhR* methylation in ALL is similar to that found for other tumor suppressors genes such as *p53, p73, p15*, and *p16* (Mulero-Navarro et al., 2006). In diffuse large B-cell lymphoma (DLBCL) cell lines, *AhR* expression was restored not only by the DNA methyl transferase inhibitor 5- Aza-2′ deoxycytidine (AZA), but also by the histone deacetylase inhibitor Trichostatin A, indicating that differences in histone acetylation may also alter *AhR* expression (Ding et al., 2015). The latter study confirmed, using knockdown experiments and those two epigenetic inhibitors, that the *AhR/ARNT* complex regulates *MEF2B* and *BCL6*. *MEF2B* codes for a key transcription factor that cooperates with co-repressors and histone-modifying enzymes to regulate gene expression while *BCL6* is a proto-oncogene selectively expressed in germinal center B-cells, in which it induces the expression of the germinal center markers *LMO2* and *MYBL1*. Thus, AhR-dependent upregulation of wild-type *MEF2B* could add an additional layer of epigenetic regulation in DLBCL besides canonical *BCL6* translocations, *BCL6* hyper-mutation and *MEF2B* mutations (Ding et al., 2015).

There are some evidences indicating that AhR and histone deacetylases become part of a common epigenetic regulatory machinery. AhR and NAD^+^-dependent deacetylase Sirtuin 1 (SIRT1) were analyzed in different disorders such as human vascular senescence and atopic dermatitis. AhR regulates cellular senescence of human endothelial cells mediated by oxidative stress. Specifically, AhR activation by indoxyl sulfate (IS) in human umbilical vein endothelial cells (HUVECs) accelerates senescence by suppressing the NAD^+^-dependent SIRT1 with a concomitant decrease in NAD^+^ levels and in intracellular nicotinamide phospho-ribosyl-transferase (iNampt) activity (Koizumi et al., 2014). These results suggest that the IS-AhR-SIRT1 pathway plays an important role in the pathogenesis of vascular senescence. AhR also appears to be a relevant regulator of the skin barrier, at least in part because of its role in xenobiotic detoxification. AhR activation by coal tar induces skin barrier repair with enhanced expression of the structural skin barrier protein filagrin (FLG) (van den Bogaard et al., 2015). As one of the available treatments for atopic dermatitis (AD), coal tar was found to promote epidermal differentiation by activating AhR. Combined activation of SIRT1 and AhR/AKT improved skin barrier repair and the therapeutic effects of coal tar on atopic dermatitis. Mechanistically, SIRT1 positively regulated AhR activation, which, in turn, enhanced AhR and AhR/AKT-induced FLG expression. SIRT1 loss in keratinocytes and mouse skin inhibits basal and ligand-induced AhR activity.

In addition to promoter hypermethylation and histone modifications, deregulation of micro-RNAs (miRNAs) as epigenetic and post-transcriptional regulators add another level of control to AhR functions in normal physiology and pathogenesis. miRNAs are small, 19-25 nucleotides long non-protein-coding RNAs that, once matured, bind to 3′-untranslated regions (3′UTR) of target messenger RNA molecules (mRNAs). miRNA binding to its cognate mRNA results in posttranscriptional suppression of gene expression through cleavage of the target mRNA molecule. miRNAs are involved in normal eukaryotic cellular functions like proliferation, differentiation, apoptosis and development and, as a result, their deregulation is associated with various diseases (e.g., in immunity, autoimmune disease, and cancer) (Lu et al., 2005). To date, the implication of the miRNA machinery in AhR-dependent signaling, and vice versa, is mostly under investigated. Original findings suggested that AhR is an important regulator of T cell differentiation (Kimura et al., 2008; Quintana et al., 2008; Veldhoen et al., 2008; Gandhi et al., 2010). In mouse models of experimental autoimmune encephalitis and collagen-induced arthritis, several laboratories have demonstrated the involvement of miRNA-based mechanisms in AhR-dependent differentiation of immune cells. In particular, it was shown that whereas Th17 differentiation via AhR depends on the miR132/212 cluster, Treg differentiation from naïve T cells by Transforming growth factor-β (TGF-β), with or without AhR ligands TCDD or 6-formylindolo[3,2-b]carbazole (FICZ), did not show significant differences in control and miR-132/212 double knock-out (DKO) mice. These data indicate that the miR-132/212 cluster participates in AhR-mediated development of Th17, but not Treg cells (Nakahama et al., 2013). Additional work has addressed the role of the AhR-inducible miR-132/212 cluster in inflammatory and ulcerative colitis in mice. The results show that AhR has specific functions in different immune cell populations: the AhR-inducible miR-132/212 cluster promoted inflammatory responses by inducing Th17 cells and suppressing the development of IL-10-producing T cells (Chinen et al., 2015). In breast cancer, miR-132, but not miR-212, inhibited cell proliferation and metastasis mediated by the *NH1* gene (*Hematological and Neurological expressed gene-1)* (Zhang et al., 2014). In contrast, AhR activation by TCDD and 3.3′-diindolylmethane (DIM) regulated miR-132/212 expression in MDA-MB-231 and T47D breast cancer cells. Chromatin immunoprecipitation (ChIP) assays demonstrated AhR binding to the two xenobiotic responsive elements (XRE) located within 1 kbp of the upstream promoter of the *miR-132/212* genes, thus supporting a direct transcriptional regulatory mechanism. Besides, inhibition of miR-212/132 in cells treated with TCDD and DIM mitigated the anti-invasive effects of these molecules, suggesting that miR-212/132 mediate, at least partially, the anti-metastatic effects of prototypical AhR ligands. Although more research needs to be done, it appears plausible that other molecules, probably other miRNAs, play a role in the anti-invasive effects of AhR. In fact, functional interactions between AhR and miR-335 have been described in breast cancer. miR-335 is an anti-metastatic miRNA that can be induced by TCDD and 6-methyl-1,3,8-trichlorodibenzofuran (MCDF) in estrogen receptor (ERα)-negative breast cancer cells. miR-335 over-expression inhibited invasion and metastasis of MDA-MB-231 LM2 cells *in vivo* (Tavazoie et al., 2008). Both TCDD and MCDF induced miR-335 and decreased *SOX-4* expression, whereas MCDF also inhibited MDA-MB-231 LM2 metastasis to the lungs in *in vivo* tail vein injection assays. The mechanistic connection between AhR and miR-335 was demonstrated by the fact that AhR knockdown abolished the effects of TCDD and MCDF on miR-335 and *SOX4* expression as well as the interaction of miR-335 with the *SOX4* 3′-UTR region (Tavazoie et al., 2008).

## New roles of AhR in physiological and pathological processes

The study of the physiological functions of AhR, and consequently, of the pathologies in which this receptor might be involved has attracted the interest of many laboratories in recent years. In addition to its well-known functions related to cell proliferation, adhesion and migration, there are other cellular processes where AhR is emerging as a relevant molecular intermediate. Among them, we will analyze here how AhR expression influences cell differentiation and pluripotency and also its probable role in maintaining a stem phenotype.

### Cell differentiation, pluripotency, and stemness

The implication of AhR in cell differentiation was described in early reports. Studies using phorbol ester as inducer, revealed that differentiation of promyelocytic HL-60 and erythroblastic HEL cells to monocytes increased the levels of transcriptionally active AhR (Hayashi et al., 1995). Moreover, treatment of pregnant mice with the AhR ligand TCDD demonstrated that this carcinogen acts directly on the embryo proper at the pre-implantation stage and that it accelerates the differentiation program during organogenesis (Blankenship et al., 1993), suggesting that AhR supports differentiation *in vivo*. In agreement with that hypothesis, recent findings have shown that AHR activation by TCDD during mouse pregnancy blocked the ability of hematopoietic stem cells (HSC) for long-term self-renewal (Laiosa et al., 2015) and that sustained AhR activation during early differentiation of mouse embryonic stem cells impairs signaling critical for the ontogeny of cardiac mesoderm and cardiomyocyte functions (Wang et al., 2016).

Genome-wide ChIP/chip analysis using wild type Hepa-1c1c7 mouse hepatocellular carcinoma cells and its AhR-deficient c37 derivative, have identified gene clusters regulated by AhR with a suspected implication in cell differentiation, patterning and development under basal cell conditions (Sartor et al., 2009). The effects of AhR on differentiation in absence of xenobiotics were also described in former reports. Experiments aiming to differentiate embryonic fibroblasts from wild type and AhR-null mice into adipocytes revealed that lack of AhR altered spontaneous differentiation and cell senescence, suggesting that AhR is an early regulator of adipocyte differentiation (Alexander et al., 1998). Accordingly, modulating AhR levels in 3T3-L1 murine fibroblasts by either receptor over-expression or knockdown showed that high receptor levels could suppress morphological adipocyte differentiation and the induction of adipogenesis-related genes, then confirming that AhR is a negative regulator of adipose differentiation of fibroblast cells (Shimba et al., 2001). But perhaps one of the fields where AhR has been more intensively scrutinized regarding differentiation is the immune system. A previous excellent review is recommended for a detail analysis of the role of AhR in immune differentiation of barrier organs such as skin, gut, and lung epithelia (Esser and Rannug, 2015). The reciprocal correlation between the generation of Treg cells expressing the transcription factor Foxp3 and pro-inflammatory T cells producing interleukin-17 (Th17) has been intensely investigated in the context of the immune system differentiation (Esser et al., 2009). Parallel reports revealed that AhR was required and regulated the differentiation of both Treg and Th17 lymphocytes *in vitro* and that such process required the presence of an externally added ligand such as FICZ or a molecule generated in the culture medium not yet identified (Quintana et al., 2008; Veldhoen et al., 2009). Latter efforts trying to identify ligands that modulate Treg and Th17 differentiation have concluded that whereas Th17 differentiation involves a XRE-mediated mechanism, Treg differentiation could take place by a non-XRE-mediated process and, therefore, that selective AhR modulators (SAhRM) could be used to modulate the Th17/Treg balance (Mohinta et al., 2015). There are evidences that kynurenine (L-Kyn), the first tryptophan metabolite of the indoleamine 2,3 dioxygenase (IDO) pathway, activates the AHR at a dose clinically relevant in humans and leads to the generation of Treg cells *in vitro*. The role for AHR in this process is supported by the fact that L-Kyn does not influence Treg generation in AHR-null T cells and it can activate the AHR-dependent transcription of classical target genes such as *Cyp1a1* and *Cyp1b1* (Mezrich et al., 2010). Moreover, AHR activation by L-Kyn regulates the expression of alternative target genes in dentritic cells including *Tgf*β*1* and *Ido1*, which, in turn, will promote the expression of anti-inflammatory genes (Nuti et al., 2014). Other potential AHR ligand is 2-(1′H-indole- 3′-carbonyl)-thiazole-4-carboxylic acid methyl ester (ITE). It promotes the induction of active immunologic tolerance by exerting direct effects on dendritic cells and T lymphocytes, being considered as a nontoxic endogenous AHR ligand potentially useful for the treatment of autoimmune disorders (Quintana et al., 2010). Interestingly, AhR can cooperate with the hypoxia-inducible factor-α (HIF-1α in the differentiation of type 1 regulatory T cell (Tr1) through metabolic reprogramming. At early stages of differentiation, HIF-1α drives Tr1 metabolic reprogramming whereas, at later times, AhR would induce HIF-1α degradation to take control of Tr1 metabolism (Mascanfroni et al., 2015). The differentiation promoting activity of AhR has been observed in additional cell types. Treatment of primary mouse keratinocytes with AhR antagonists CH223191 and GNF351 impaired their terminal differentiation while transcriptomic analysis of keratinocytes isolated from *AhR*+*/*+ and *AhR-/-* mice has generated a differential gene expression pattern in which terminal differentiation genes were significantly inhibited in *AhR-/-* keratinocytes (van den Bogaard et al., 2015). However, AhR does not seem to have a general contribution to immune and T cell differentiation since it is not seem to be required for the differentiation of T cytotoxic 17 (Tc17) cells (Hayes et al., 2014). Although AhR has a pro-differentiation role in the majority of cell types investigated, a recent study indicated that receptor activation by TCDD inhibited proliferation and differentiation of pre-osteoblasts MC3T3-E1 cells in a dose-dependent manner, and that pretreatment with the AhR antagonist CH-223191 restored the differentiation potential of MC3T3-E1 cells (Yu et al., 2014). Thus, it appears that AhR has cell-type specific effects on cell differentiation, as it is already known with respect to cell proliferation and migration (Pohjanvirta, 2012).

It appears evident that AhR is relevant for cell differentiation. Recent reports are also showing that transposable elements can regulate embryonic cell differentiation. Since AhR controls the expression of murine and human retrotranspons, the link between AhR, retrotransposons and cell differentiation seems likely. In addition to our previous work demonstrating that differentiation of human carcinoma NTERA-2 cells can be induced by an AHR-regulated *Alu* retrotransposon (Morales-Hernández et al., 2016), additional studies (see Section AhR in the Control of Gene Expression through Mobile Genetic Elements above) have identified a *LINE-1* element that responds to AhR ligands during the differentiation of renal carcinoma cells (Ramos et al., 2011) and a *LINE-1* that can retrotranspose upon AhR activation by food-borne mutagens (Okudaira et al., 2013). It will be important to establish whether AhR has a more general role in the control of transposable elements known to contribute to cell differentiation. A complementary view to this question is to search for transposable elements that may regulate pluripotency and stemness through the AhR. Although this issue remains to be addressed in detail, AhR is connected to cell stemness since its expression is transcriptionally repressed in mouse embryonic stem cells by a signaling pathway involving pluripotency factors OCT3/4, NANOG, SOX2 and polycomb proteins. Such repression is reversible so that rapid AhR upregulation may trigger embryonic stem cell differentiation (Ko et al., 2013). In addition, the anti-allergy drug tranilast can revert differentiation during the reprogramming of mouse embryonic fibroblasts to induced-pluripotent stem (iPS) cells by the AhR-dependent control of miR-302 (Hu et al., 2013). Transposons are also coupled with pluripotency and stemness. Upregulation of *Alu* retrotransposons in human adult stem cells results in nuclear cytotoxicity associated to permanent DNA damage, loss of efficient DNA repair and senescence; *Alu* elements may have a causal role in the process because its downmodulation reverses the senescent phenotype and rescues self-renewal and the expression of pluripotency regulators (Wang et al., 2011). Retrotransposon-derived transcripts are also relevant in the maturation of germinal cells, in particular of spermatocytes. Piwi-interacting RNAs (piRNAs) produced from transposons are responsible for the degradation of mRNAs and long non-coding RNAs (lncRNAs) in late spermatocyte differentiation, supporting that retrotransposons influence pluripotency and maturation of the male germline (Watanabe et al., 2015). High throughout analysis of the nuclear and cytosolic transcriptome of human and mouse stem cells identified a novel class of stem cell-specific transcripts, including long terminal repeat (LTR)-derived transcripts, presumably associated to gene enhancer regions relevant for pluripotency (Fort et al., 2014). In agreement with that possibility, it has been observed that certain endogenous retroelements were markedly overexpressed whereas others were repressed during the reprogramming of mouse embryonic fibroblasts, human CD34+ cells and human primary hepatocytes to iPS cells. To some extent, that expression pattern of retrotransposon expression found in iPS cells resembles that found in embryonic stem cells. Importantly, during reprogramming and in established iPS cells, the upregulation of specific retrotransposons significantly influenced the expression of nearby genes, thus supporting a gene regulatory function for those repetitive elements in pluripotency and stemness (Friedli et al., 2014). Altogether, we propose the existence of a functional link between AhR and differentiation and between differentiation and pluripotency with transposable elements. Notably, studies are starting to emerge establishing a signaling pathway that connects AhR with differentiation/pluripotency and both with retrotransposon expression. This is not surprising considering the very probable relevance of mobile genetic elements in the control of cell function.

### Deregulation of AhR-related cellular and tissue differentiation

AhR has a ubiquitous cell and tissue distribution and it is highly conserved in evolution, which supported its implication in different physiological processes (Section Cell Differentiation, Pluripotency, and Stemness). Consequently, alterations in AhR-dependent signaling may cause different developmental and homeostatic disorders in the organism. In this review, we will focus in alterations affecting the cardiac, hematopoietic, epithelial and hepatic systems.

The fact that AhR has a relevant contribution to the control of cell differentiation under physiological conditions suggests that it may also be important for differentiation-related pathologies. In a panel of metastatic and invasive human breast cancer cell lines, AhR activation by exogenous ligand blocked their anchorage-independent growth and metastatic potential while promoting their differentiation to a less invasive phenotype. Consistently, AhR knockdown impaired the protective effects of receptor expression and rescued cell motility and invasion, suggesting that AhR-induced differentiation could be a valuable tool in breast cancer treatment (Hall et al., 2010). Neuroblastoma is among the most common malignant diseases of the infancy. The comparative analysis of neuroblastoma tumors having or lacking amplification of the prognostic MYCN marker revealed that AhR expression was inversely correlated with MYCN amplification and, importantly, with an increased grade of tumor differentiation. Gene expression analysis identified MYCN as a novel AhR target gene repressed in neuroblastoma cells by a mechanism involving the transcription factor E2F1 (Wu et al., 2014). AhR is also involved in leukemia cell differentiation. HL-60 human myeloblastic leukemia cells have been used as a model because they can be readily differentiated by retinoic acid (RA) treatment. RA increased AHR levels during HL-60 differentiation, in parallel with a reduction in the levels of the pluripotency and stem cell marker OCT4. The implication of AHR in the mechanism was confirmed by the fact that receptor overexpression inhibited *OCT4* and *ALDH1* levels and exacerbated RA-induced granulocytic differentiation of HL-60 cells as determined by the upregulation of CD38 and Cd11b markers (Bunaciu and Yen, 2011). Furthermore, the same laboratory also demonstrated that AHR activation by the non-toxic FICZ ligand enhanced RA-induced granulocytic differentiation of HL-60 cells likely through the RAF-MEK-ERK signaling pathway and downstream targets. In agreement, RA and FICZ co-treatment increased AHR levels and its transcriptional activity for typical target genes such as *CYP1A2* and *p47phox* (Bunaciu and Yen, 2013). The AHR agonist VAF347 also increased receptor levels in RA-treated HL-60 cells concomitantly with their differentiation to a granulocytic lineage; further supporting that AHR upregulation in leukemia cells favors a differentiated phenotype (Ibabao et al., 2015). VAF347 was also able to promote the differentiation of IL-22-producing Th cells from naïve CD4+ Th cells, suggesting that AhR activation could increase the beneficial effects of IL-22 while preventing the secretion of pro-inflammatory cytokines (Baba et al., 2012).

We have recently described that the guanine nucleotide exchange factor Vav3, a protooncogene that regulates the activation of Rho and Rac GTPases, is an AhR transcriptional target in murine embryonic fibroblasts. Notably, AhR-deficient mice develop respiratory and cardiovascular phenotypes that resemble those of *Vav3-/-* mice including hypertension, tachypnea, and sympathetic excitation. Similarly to Vav3-deficient mice, *AhR-/-* animals develop cardiovascular remodeling characterized by concentric left ventricular hypertrophy, thickening of the arterial wall, increased numbers of vascular smooth muscle cells in arterial walls and extensive fibrosis in the heart and kidneys (Sauzeau et al., 2011). Earlier studies did also show that *AhR-/-* mice had cardiac hypertrophy, blood pressure overload and elevated levels of the proteins endothelin 1 (ET-1), angiotensin-2, β-myosin heavy chain and atrial natriuretic factor (Lund et al., 2003). Interestingly, the higher risk for developing cardiovascular pathologies in chronic kidney disease patients could be due to exposure to uremic toxins derived from tryptophan metabolism. Tryptophan metabolites could in turn activate AhR and induce the generation of reactive oxygen species contributing to leukocyte activation and inflammation (Sallée et al., 2014).

The ability to self-renew, and the balance between the pool of HSCs and the different subsets of differentiated hematopoietic progenitors are controlled by a tightly regulated mechanism. AhR is acquiring an emerging role in the deregulation of these processes. Prolonged loss of AhR results in sensitivity to stress and in premature exhaustion and aberrant changes in HSCs that develop into a myeloproliferative disorder in aged mice (Singh et al., 2014). Altered AHR expression and activity have also been described in human leukemia and lymphoma cells (Hayashibara et al., 2003; Mulero-Navarro et al., 2006). Additional work has identified intermediate proteins of the AhR signaling pathway that are rapidly and robustly activated by small molecules (SR1, LGC006, UM729) in normal and leukemic stem cells in culture (Boitano et al., 2010; Pabst et al., 2014). In acute myeloid leukemia (AML) cells, the CD34^+^CD15^−^ population increases following AhR suppression by a block in differentiation rather than by an increase in proliferation. Moreover, the levels of CD34^+^CD38^−^ and CD34^+^CD38^+^ compartments benefitted from the presence of the SR1 and UM729 molecules with respect to untreated cultures (Pabst et al., 2014). Dendritic cells (DC)-based immunotherapy is actually considered an important tool in the treatment of patients. Tumor cells, myeloid-derived suppressor cells and tumor-associated macrophages keep a potent immunosuppressive environment to avoid DC maturation. The acquisition of maturation-associated genes, and their contribution to the maintenance of an immature state of monocyte-derived dendritic cells (MDDCs) and myeloid DCs are partly dependent on AhR activity. In fact, the transcriptional activity of AhR appears to be involved in the ERK-dependent profile of gene expression in MDDC cells. ERK up-regulates CCL2 expression, a chemokine constitutively produced by immature MDDCs, while impairing the expression of maturation markers of dendritic cells such as RUNX3, ITGB7 and IDO1 (Aguilera-Montilla et al., 2013). Furthermore, the MEK-ERK signaling pathway also regulates antigen capture and lymph node homing. MEK inhibition likely increases AhR transcriptional activity in MDDC cells because the AhR partial antagonist α-naphtoflavone (α-NF) impaired U0126-induced gene up-regulation. Moreover, U0126 treatment led to *in vivo* occupancy of AhR-binding sites followed by a reduction in total AhR protein levels, suggesting the generation of a pool of transcriptionally active receptor by MEK inhibition. The immune system is very sensitive to AhR-mediated toxicity and responds to particularly low concentrations of chemicals (Kerkvliet et al., 2002), thus protecting the organism against different insults including toxins. AhR participates in Th17 cell differentiation through the regulation of Stat1, with whom it forms a complex in macrophages to negatively regulate NF-κB-mediated pro-inflammatory responses. Besides, AhR interacts with c-Maf to promote the differentiation of type 1 regulatory T cells induced by IL-27. The compiled data suggest that AhR plays multiple roles in distinct immune cell populations (Apetoh et al., 2010).

Chloracne is a skin disease that can be induced by TCDD in humans. The molecular mechanism driving chloracne is AhR-related and it seemingly involves both stem cells and keratinocytes through a terminal differentiation process activated by filaggrin (Sutter et al., 2011). Moreover, the skin inflammatory response known as contact dermatitis is triggered by keratinocytes in which AhR is constitutively active. In this regard, the major role of AhR in skin pathologies could be to modulate the expansion of initiated stem cells and keratinocytes that generate anti-apoptotic signals (Ikuta et al., 2009). In human melanoma, AhR regulates aldehyde dehydrogenase to block melanoma tumorigenesis and metastasis (Contador-Troca et al., 2015). We have recently reported that stable AhR knockdown in B16F10 mouse melanoma cells enhanced their primary tumorigenicity and their metastatic potential to the lungs, whereas constitutive AhR activation strongly blocked melanoma progression. Interestingly, AhR knockdown increased melanoma cell migration and invasion and the expression of mesenchymal markers α-smooth muscle actin (α-SMA) and Snail. Moreover, the pro-tumoral phenotype caused by AhR depletion in the tumor cell (cell autonomous) required AhR expression in the microenvironment as *AhR-/-* mice could not support tumor growth and metastatization of AhR-depleted cells (Contador-Troca et al., 2013).

There is considerable evidence to support the critical role played by AhR and AhR-regulated genes in human and murine hepatocarcinogenesis. For instance, it has been shown that hexachlorobenzene (HCB) induces the expression of AhR in rat liver pre-neoplastic foci and in human hepatocarcinoma HepG2 cells (de Tomaso Portaz et al., 2015). AhR activation is associated with the disruption of cell-cell contacts in liver stem-like WB F344 cells, suggesting that it could contribute to the release of liver metastasis (Köhle et al., 1999; Weiss et al., 2008). Accordingly, TCDD releases confluent WB-F344 cells from contact inhibition and stimulates cell growth via activation of Jun D and Cyclin A (Weiss et al., 2008). In agreement with a positive effect of AhR in liver growth, *AhR-/-* mice show reduced liver size, portal fibrosis and impaired induction of detoxifying enzymes that could compromise its function (Fernandez-Salguero et al., 1995; Peterson et al., 2000; Corchero and Fernández-Salguero, 2005). Some of these phenotypes in AhR-null livers appear to be related to the over-activation of TGFβ due to the up-regulation in the extracellular matrix of its latency protein LTBP-1, which was in fact identified as a novel AhR target gene (Santiago-Josefat et al., 2004; Corchero and Fernández-Salguero, 2005; Gomez-Duran et al., 2006, 2008). The scenario in the liver appears more complex since AhR also regulates RA levels and retinoid homeostasis (Andreola et al., 1997, 2004), which are functionally related to TGFβ and to cell growth and differentiation. Further work is needed to clearly define the pathological consequences of altered AhR expression in the liver. Recently, the role of AhR in the differentiation process of hepatic stem/progenitor cells (HpSC) has been regarded as a new important aspect in receptor biology. The effects of AhR on hepatic stem cells has been studied by Harril and coworkers after defining successful culture conditions for rat hepatic stem cells treated with hyaluronans, TCDD, FICZ and DIM. They showed that chronic administration of TCDD to female rats produced pathological changes that included hepatocellular hypertrophy and proliferation of HpSC (Harrill et al., 2015).

## Medical approach of AHR

In this section, we will discuss possible AhR ligand candidates and AhR itself both as a potential therapeutic target and as a beneficial molecule whose regulation could improve human health status.

### AhR as a therapeutic target in human disease

The identification of AhR ligands with well-described health-promoting effects and/or beneficial pharmaceutical properties has prompted research on the development of drugs to target AhR for the treatment of diseases including specific tumors, immune disorders, inflammatory disease and to enhance the production of hematopoietic stem cells. The development of drugs that target AhR is based on selective AhR modulators (SAhRMs), in which a ligand exhibits tissue-specific AhR agonistic or antagonistic activity (Jin et al., 2012). Tissue-specific differences in agonistic or antagonistic activity of a ligand are due to multiple factors including ligand-induced conformational changes in the receptor and in subsequent interactions with critical co-activators and co-repressors. Various classes of AhR ligands, and different molecular species within the same class, can differentially modulate AhR activity causing different gene expression outcomes. For this reason, AhR may become a potentially important drug target that can be efficiently regulated in a cell type-specific manner.

As AhR is highly expressed in a wide panel of tumors, molecules with antagonistic AhR activity could be considered potential candidates for the treatment of such diseases. The best known antagonist of AhR is alpha-naphthoflavone, although it also has weak agonist activity at relatively high concentrations (Gasiewicz and Rucci, 1991). A high-affinity AhR antagonist, StemRegenin 1 (SR1), has been recently developed that increases the proliferation of human hematopoietic stem cells *in vitro* (Boitano et al., 2010). Interestingly, culture with SR1 had no effect on murine HSCs but expanded the pool of CD34^+^ cells from the bone marrow of humans, monkeys (cynomologous and rhesus), and dogs. Potent effects of the candidate AhR ligand bilirubin (Kerkvliet, 2009) and ITE (Nuti et al., 2014) on the immune system have also been reported. In particular, treatment of mice with bilirubin suppressed the development of autoimmune disease whereas depletion of endogenous bilirubin significantly exacerbated the disease (Kerkvliet, 2009). The administration of synthetic ITE reduces OCT4 levels and induces the differentiation of stem-like cancer cells, a process that impairs their tumorigenic potential in both subcutaneous and orthotopic xenograft tumor models (Cheng et al., 2015). The novel roles of tryptophan derivatives (e.g., ITE), together with the likely implication of the AhR pathway in regulating cell stemness, open interesting new therapeutic avenues to target stem-like cancer cells. Moreover, in ovarian cancer, it has been demonstrated that ITE regulates cancer cell proliferation and migration via AhR using *in vitro* and/or *in vivo* models. This potential anti-ovarian cancer activity of AhR could help optimize strategies for ovarian cancer therapy (Wang et al., 2013).

### Benefits from the use of AhR ligands in a balanced diet

Among the numerous plant-derived nutrients and phyto-chemicals present in the human diet, flavonoids are the most abundant and are ubiquitous in fruits, vegetables, tea and wine. Interestingly, the quercetin, apigenin and kaempferol present in certain foods (e.g., rosehip, linden flower, honey, grapes, and tarhana) mediate an agonist/antagonist synergistic effect of AhR activity in different cell types (Van der Heiden et al., 2009). Furthermore, many flavonoids have diverse health promoting effects, including anti-allergic and anti-inflammatory activities (Kawai et al., 2007; Benavente-García and Castillo, 2008). In addition, the dietary flavonoid naringenin induces the generation of regulatory Treg cells by an AhR-mediated pathway (Wang et al., 2012). Recent studies have also shown that dietary indoles suppressed hypersensitivity by inducing a switch from pro-inflammatory Th17 to anti-inflammatory Treg cells through the generation of a specific miRNA profile (Singh et al., 2016). Interestingly, the flavone-based antagonist resveratrol has gained considerable interest due to its apparent beneficial effects on human health. Resveratrol inhibited transcription of the target gene *CYP1A1 in vitro* by preventing AhR activation (Ciolino et al., 1998). Importantly, indole glucosinolates present in cruciferous vegetables are degraded to indole-3-carbinol (I3C) and indolo[3,2*b*]carbazole, the latter having high AhR affinity (Bjeldanes et al., 1991). As a nutritional supplement, daily administration of I3C, at doses of 400 mg and 800 mg, was well tolerated in a phase I clinical trial in 17 premenopausal women (Reed et al., 2005). Probiotic bacteria and yeast belonging to the microflora of the human intestine and skin are able to produce AhR ligands (e.g., indole-3-aldehyde, indirubin) intended to enhance the AhR-mediated barrier function (Magiatis et al., 2013; Fukumoto et al., 2014). Bilirubin is an antioxidant AhR ligand that can activate UDP glucuronosyltransferase 1A1 (UGT1A1) in a negative auto-regulatory feedback loop (Togawa et al., 2008). Maternal exposure to AhR agonists has been proposed as a risk factor for breast cancer in the offspring through epigenetic inhibition of the tumor suppressor BRCA-1 (Breast cancer 1), whereas dietary AhR antagonists may exert protective effects against such disease (Papoutsis et al., 2015).

## Conclusions and future perspectives

A main conclusion that can be obtained from recent studies dealing with the implication of the dioxin receptor in physiology is that this receptor is involved in significantly more cellular processes that initially anticipated. It is clearly established that AhR is required for an ample set of toxicological and carcinogenic responses against environmental compounds. Its relevant contribution to the regulation of signaling pathways that control cellular functions such as proliferation, adhesion and migration is also generally accepted. AhR is known to participate in the homeostasis of different tissues and organs and in the pathological processes that result from their dysfunction. The critical importance of the balance between cell differentiation and pluripotency has attracted a great deal of attention toward AhR. The immune system, epithelia from different embryonic origins, the germline, adipose tissue and bone cells have been used to conclude that, with some exceptions, the AhR promotes differentiation. The use of murine and human experimental models in which AhR expression has been depleted confirms the induction of an undifferentiated and more pluripotent phenotype. AhR may modulate cell differentiation by acting as a direct transcription factor, but also by regulating the expression of retrotransposons whose transcripts would finally repress stemness genes. This represents a novel and more complex mechanism of gene regulation that transformed cells could use to scape differentiation through AhR inhibition. Since altered AhR expression can be associated to human disease, it is reasonable to propose that the design of specific and selective AhR modulators may have therapeutic value in the treatment of pathologies such as undifferentiated and highly malignant tumors. Another point of interest relates to the inclusion in the diet of molecules acting through AhR that could contribute to a better health status by preventing undesired events of dedifferentiation and the acquisition of a stem-like phenotype leading to cell transformation. Future work will probably pursue the identification of molecular intermediates and of yet undetermined signaling pathways in which AhR could have a central role in the equilibrium between differentiation and pluripotency. Presumably, the investigation of mobile repetitive elements will play a significant part in this field.

## Author contributions

SM and PF designed the contents of the article and wrote the review.

## Funding

This work was supported by grants to PF from the Ministerio de Economía y Competitividad (BFU2011-22678 and SAF2014-51813-R), the Junta de Extremadura (GR10008 and GR150088) and the Red Temática de Investigación Cooperativa en Cáncer (RTICC), Carlos III Institute, Ministerio de Economía y Competitividad (RD12/0036/0032). All the Spanish funding is co-sponsored by the European Union FEDER program.

### Conflict of interest statement

The authors declare that the research was conducted in the absence of any commercial or financial relationships that could be construed as a potential conflict of interest. The Associate Editor JL declares that, despite having collaborated with the author PF, the review process was handled objectively and no conflict of interest exists.
